# A simulation study of a honeybee breeding scheme accounting for polyandry, direct and maternal effects on colony performance

**DOI:** 10.1186/s12711-021-00665-8

**Published:** 2021-09-08

**Authors:** Tristan Kistler, Benjamin Basso, Florence Phocas

**Affiliations:** 1 Université Paris-Saclay, INRAE, AgroParisTech, GABI, 78350 Jouy-en-Josas, France; 2 INRAE, UR 406 Abeilles et Environnement, 84914 Avignon, France

## Abstract

**Background:**

Efficient breeding programs are difficult to implement in honeybees due to their biological specificities (polyandry and haplo-diploidy) and complexity of the traits of interest, with performances being measured at the colony scale and resulting from the joint effects of tens of thousands of workers (called direct effects) and of the queen (called maternal effects). We implemented a Monte Carlo simulation program of a breeding plan designed specifically for *Apis mellifera*’s populations to assess the impact of polyandry versus monoandry on colony performance, inbreeding level and genetic gain depending on the individual selection strategy considered, i.e. complete mass selection or within-family (maternal lines) selection. We simulated several scenarios with different parameter setups by varying initial genetic variances and correlations between direct and maternal effects, the selection strategy and the polyandry level. Selection was performed on colony phenotypes.

**Results:**

All scenarios showed strong increases in direct breeding values of queens after 20 years of selection. Monoandry led to significantly higher direct than maternal genetic gains, especially when a negative correlation between direct and maternal effects was simulated. However, the relative increase in these genetic gains depended also on their initial genetic variability and on the selection strategy. When polyandry was simulated, the results were very similar with either 8 or 16 drones mated to each queen. Across scenarios, polyandrous mating resulted in equivalent or higher gains in performance than monoandrous mating, but with considerably lower inbreeding rates. Mass selection conferred a ~ 20% increase in performance compared to within-family selection, but was also accompanied by a strong increase in inbreeding levels (25 to 50% higher).

**Conclusions:**

Our study is the first to compare the long-term effects of polyandrous versus monoandrous mating in honeybee breeding. The latter is an emergent strategy to improve specific traits, such as resistance to varroa, which can be difficult or expensive to phenotype. However, if used during several generations in a closed population, monoandrous mating increases the inbreeding level of queens much more than polyandrous mating, which is a strong limitation of this strategy.

**Supplementary Information:**

The online version contains supplementary material available at 10.1186/s12711-021-00665-8.

## Background

Implementing an efficient breeding program for *Apis mellifera* is difficult because of its genetic and reproductive specificities, and of the complexity of its traits of interest, which are measured in the hive at the colony scale. A colony is composed of three types of individuals: a fertilized queen and her progeny composed of tens of thousands of female workers and a highly variable number of male drones, generally in the hundreds or thousands during mating periods. Workers contribute to the collection of pollen and nectar, the production of honey, wax or royal jelly, and the nursing of the queen, but they do not play a role in reproduction. On the contrary, drones serve mainly for reproduction. The queen has both a role in production since she has a strong influence on workers in terms of egg-laying rate and pheromone release, and a role in reproduction since it is the only fertile female of the colony. In addition, honeybee is a haplodiploid species, with queens and workers being diploid and drones being haploid. Males develop from unfertilized oocytes that are produced by arrhenotokous parthenogenesis whereas females develop from fertilized oocytes. This strategy of reproduction plays a major role in the evolution of Hymenoptera, and one of its advantages is that it efficiently eliminates deleterious recessive alleles present at the hemizygous state in males [1]. However, when exposed to stress conditions which increased inbreeding, haplodiploid populations can be prone to faster decline or extinction than diploid populations [2]. Some of these mechanisms of extinction are linked to the sex determinism of Hymenoptera species, among which, the honeybee. The sex of honeybees is determined by the complementary sex determiner (*csd*) gene [3–5] with over one hundred alleles currently identified [6]. Heterozygosity at this sex locus results in diploid workers or queens and homozygosity results in diploid drones, which are withdrawn by the workers at the larval stage [7].

Another specificity of honeybees is the polyandry of the queen, which reduces the risk of co-occurrence of functionally identical *csd* alleles in the progeny. Within a few days to a few weeks after emergence, an unrestricted queen makes one to five mating flights during which she mates with on average 12 to 16 drones [8–12] in congregation areas. The queen stores within a few days all the sperm she will use during her lifetime. Such congregation areas bring together mates from several kilometers around.

Breeding programs, which require controlled mating, often rely on the use of artificial insemination or isolated mating stations to produce daughters from both male and female brood stocks with high breeding values. On mating stations, breeders can restrict the presence of drone-producing queens (DPQ) and virgin queens to those with the desired genetics. Higher control on the origin of the semen that fertilizes a queen is possible when artificial insemination is used; for instance, semen can originate from a single drone or from the mixing of sperm from multiple drones bred by a single DPQ [13]. Monoandrous mating can facilitate the phenotyping of colony traits that are difficult to measure when several patrilines coexist in the worker population [14, 15], and help to better discriminate each parent’s contribution to the observed phenotypes. However, single-drone inseminated queens generally have a shorter life expectancy and do not form as vigorous colonies as polyandrous mated queens [14], mainly because the spermatheca is insufficiently filled [16]. To solve this issue, the practice of single-drone insemination is mainly applied in a few honeybee breeding programs with short maternal generation intervals [17].

Another limiting aspect of monoandrous mating is due to the resulting increased inbreeding and genetic drift, and thereby to the loss of diversity in *csd* alleles, which may lead to weaker colonies with less honey-producing capabilities because many larvae are lost [18, 19].

To quantify the impact of inbreeding on long-term efficiency of honeybee breeding programs, Moritz [20] derived the expected selection responses and levels of inbreeding based on a deterministic prediction model that compared two different mating strategies: mass or within-family selection. In the comparison of Moritz [20], queens and drones were reared from all selected colonies in the test population that was subdivided into families of equal size. The semen of all selected drones was pooled and each selected queen was inseminated with semen from the semen pool. Decreasing selection pressures from one year to the next were considered to maintain low inbreeding rates while obtaining an optimal selection response for a given time horizon. Moritz quantified the inbreeding depression by applying a regression coefficient of colony performance on inbreeding level. This is a major drawback of Moritz’s study since the results of the simulation depend on a single empirical estimate (taken from [21] as cited in [20]) of the inbreeding depression effect, which was obtained for specific environmental conditions on few colonies. Moritz showed that mass selection was more efficient than within-family selection, unless the target time horizon covered more than 20 generations and proposed to use mass selection whenever possible but to keep the average within-colony inbreeding coefficient below a critical threshold of 25%.

In another deterministic simulation study, Omholt and Adnoy [22] predicted the effects of various breeding strategies on colony performance and on the frequency of diploid drones. They showed that the highest genetic gain but also the highest increase in consanguinity and in frequency of diploid drones were obtained when queens were selected among the whole population without any consideration of their pedigree as opposed to a within-maternal line selection (with one dam replacement per sib-group).

However, these two early studies made two major simplifications. First, they considered the quantitative trait of interest as resulting only from the queen’s genotype, thus ignoring the direct effect of workers on the colony performance, which for half of its value is due to the DPQ. Second, they derived the same average inbreeding level for all the queens produced in a given year and ignored the strong sampling variations that occur in small breeding populations. Therefore, further work is needed to design optimal honeybee breeding programs under a broad range of breeding strategies and based on stochastic simulations to better account for the limited size of usual honeybee breeding populations.

In a recent simulation study, Plate et al. [23] compared the genetic gains and variances that account for the effects of both the queen and workers on the colony performance under the assumptions of either the infinitesimal genetic model or a finite genetic model with 200 or 400 unlinked loci to describe the genetic variation underlying a quantitative trait. They observed similar results for the two genetic models over the first 20 years of selection in a closed population, but for a time horizon of 100 years, they found a twofold more drastic loss of genetic variance in the finite loci models than in the infinitesimal model. Considering the finite loci model [24], they also explored the impact of the number of influential loci, the population size and the selection rates. However, the basic assumption that a constant set of a few hundred unlinked loci plays the same role over such a long-time horizon does not seem biologically well-founded. In addition, it is very unlikely that the same breeding strategy will be applied over such a long period.

In all these previous simulation studies of honeybee breeding schemes, polyandry was the only mating system considered. Nevertheless, over the last 20 years there has been increasing interest to consider inseminations from single drones in honeybee selection programs, as popularized by Harbo [14], among others.

Therefore, in our study, we developed a Monte Carlo simulation program of a honeybee breeding scheme in which selection was based on colony performance during a time horizon of 20 years and under the assumption of an infinitesimal genetic model that accounts for both the direct effects of the workers and the maternal effect of the queen on the colony performance. Our breeding design enabled us to compare mass *versus* within-family selection, the latter not being previously studied by Plate et al. [23, 24]. Assuming that the semen came from a single DPQ for each inseminated queen, the objective of our study was to quantify the impact of monoandrous *versus* polyandrous mating on colony performance, inbreeding level and genetic gains depending on the selection strategy considered, i.e. mass or within-family selection.

## Methods

### Phenotype modelling

**Fig. 1:**
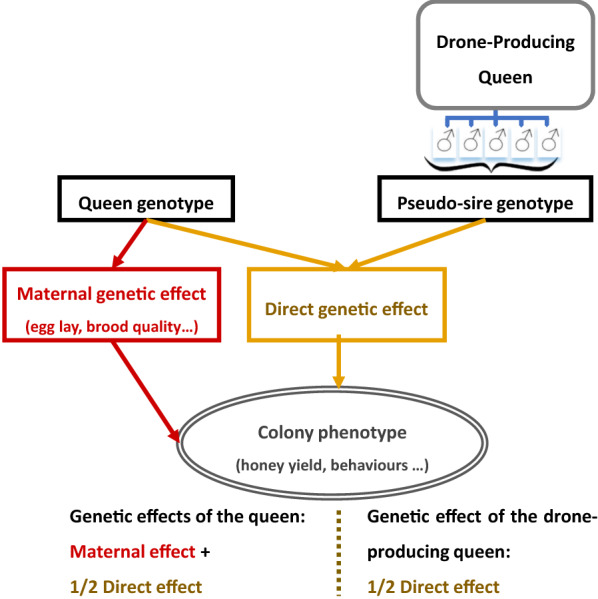
Description of the complex phenotype of a honeybee colony. The complex phenotype of the colony results from the activity of the queen and all the workers whose genotypes come from both the queen and the drone-producing queen (considered as a pseudo-sire, i.e. a virtual diploid sire)

Figure 1 represents the complex phenotype resulting from the activity of the queen and all the workers of the colony, their genotypes coming from both the queen and the DPQ, which is viewed as a virtual diploid sire.

A honeybee colony phenotype can be considered to be the sum of the contributions of the maternal genetic effects of the queen (Q), the mean direct genetic effect of the workers’ (W), and the environmental effects on the colony performance [25, 26]. This phenotype modelling was used to describe the performance (P) of a honeybee colony quantitative trait under the assumption of an infinitesimal polygenic model (as defined by Fisher [27]) accounting for maternal effects [28]. In this model, the performance of a colony results from the maternal genetic effect of the queen and the direct genetic effect of the workers. Thus, P results from the sum of $BV_{mat}^{Q}$ (the maternal breeding value of the queen) and $\overline{{BV_{dir}^{W} }}$ (the average direct breeding value of the workers’ group) of a colony, plus a non-heritable residual effect $e$, so that:

1 $$P = BV_{mat}^{Q} + \overline{{BV_{dir}^{W} }} + e.$$

The residual variable was calculated as a realization of $N\left( {0,\sigma_{e}^{2} } \right)$ with $\sigma_{e}^{2} = 30$. This performance variable was used as the selection criterion. We did not use best linear unbiased prediction (BLUP)-estimated breeding values, which consider the performances of relatives.

### Breeding values in the base population and inheritance

Direct and maternal breeding values of the base female population (generation t = 0) followed a bivariate normal distribution with expectation 0 and variance–covariance matrix ${{\varvec{\Sigma}}}_{{{\varvec{\tt{BV}}}}}^{{\mathbf{2}}}$:

$${{\varvec{\Sigma}}}_{{{\mathbf{BV}}}}^{{\mathbf{2}}} = \left( {\begin{array}{*{20}c} {\sigma_{{BV_{dir} }}^{2} } & {\sigma_{{BV_{dir} , BV_{mat} }} } \\ {\sigma_{{BV_{dir} , BV_{mat} }} } & {\sigma_{{BV_{mat} }}^{2} } \\ \end{array} } \right),$$

where $\sigma_{{BV_{dir} }}^{2}$ and $\sigma_{{BV_{mat} }}^{2}$ are the variances of the direct and maternal breeding values of the base queens, respectively, and $\sigma_{{BV_{dir} , BV_{mat} }}$ is the covariance between direct and maternal breeding values of the base queens.

Transmission of breeding values to the next generation was modeled for queens, drones and worker groups. We define ${\mathbf{BV}}$ as the vector (${\mathbf{BV}}_{{{\mathbf{dir}}}} , {\mathbf{BV}}_{{{\mathbf{mat}}}}$), in which ${\mathbf{BV}}_{{{\mathbf{dir}}}}$ and ${\mathbf{BV}}_{{{\mathbf{mat}}}}$ are, respectively, the vectors of all the queens’ direct (dir) and maternal (mat) breeding values ($\tt BV$) in the population. Superscripts are added to ${\mathbf{BV}}$ to indicate to which individuals, or group of individuals, it refers. For example, ${\mathbf{BV}}^{{\mathbf{Q}}}$ corresponds to the ${\mathbf{BV}}$ of queens. Fisher’s inbreeding coefficient is noted *F* and was obtained using a tabular method to calculate a haplodiploid relationship matrix as described by Fernando and Grossman [29], which is further described below.

The Mendelian sampling term ${\varvec{\upvarphi }}$ is defined as the difference between an offspring’s breeding value and its parents’ mean breeding value. ${\varvec{\upvarphi }}$ represents the vector of direct and maternal sampling terms $(\upvarphi_{{{\text{dir}}}} , \upvarphi_{{{\text{mat}}}} )$ and is sampled from a bivariate normal distribution $N\left( {{\mathbf{0}},{{\varvec{\Sigma}}}_{{{\mathbf{meiosis}}}}^{{\mathbf{2}}} } \right)$ with ${{\varvec{\Sigma}}}_{{{\mathbf{meiosis}}}}^{{\mathbf{2}}} { = }\frac{{1}}{{4}} \cdot \left( {{1} - F} \right) \cdot {{\varvec{\Sigma}}}_{{{\mathbf{BV}}}}^{{\mathbf{2}}}$ with $F$ being the inbreeding coefficient of the offspring’s dam. A superscript indicates if the Mendelian sampling term comes from the gametes of a DPQ or a breeding queen (BQ).

Drones inherit their breeding value only from their dam, which is a DPQ, as formulated in Eq. (2):

2 $${\mathbf{BV}}^{{\mathbf{D}}} = \frac{1}{2}{\mathbf{BV}}^{{{\mathbf{DPQ}}}} + {\varvec{\upvarphi }}^{{{\mathbf{DPQ}}}} .$$

Queens inherit their ${\mathbf{BV}}$ from a breeding queen and a drone (D), which is randomly chosen among those having mated with their dam. The Mendelian sampling term ${\varvec{\upvarphi }}$ of a queen is only due to the dam, since drones produce their gametes by mitosis. Therefore, the breeding value of a queen is derived as:

3a $${\mathbf{BV}}^{{\mathbf{Q}}} = \frac{1}{2} \cdot {\mathbf{BV}}^{{{\mathbf{BQ}}}} + {\mathbf{BV}}^{{\mathbf{D}}} + {\varvec{\upvarphi }}^{{{\mathbf{BQ}}}} .$$

Alternatively, the breeding value of a queen can also be described at the scale of the diploid parents, by replacing ${\mathbf{BV}}^{{\mathbf{D}}}$ in Eq. (3a) by Eq. (2):

3b $${\mathbf{BV}}^{{\mathbf{Q}}} = \frac{1}{2}\left( {{\mathbf{BV}}^{{{\mathbf{BQ}}}} + {\mathbf{BV}}^{{{\mathbf{DPQ}}}} } \right) + {\varvec{\upvarphi }}^{{{\mathbf{BQ}}}} + {\varvec{\upvarphi }}^{{{\mathbf{DPQ}}}} .$$

Workers, which are present in large numbers in the colony, are considered by a mean effect. This workers group’s $\overline{{{\mathbf{BV}}}}$ is described in Eq. (4) as the sum of half their queen’s ${\mathbf{BV}}$ and the mean ${\mathbf{BV}}$ of $n_{D}$ drones that mated with this queen, assuming a balanced contribution of each drone having inseminated a queen to its workers descendance. No Mendelian sampling term was considered for the workers group, since the mean Mendelian sampling term of this group tends toward 0 because it is the mean over thousands of Mendelian sampling terms centered on 0:

4a $$\overline{{{\mathbf{BV}}^{{\mathbf{W}}} }} = \frac{1}{2} \cdot {\mathbf{BV}}^{{\mathbf{Q}}} + \frac{1}{{n_{D} }}\mathop \sum \nolimits_{k = 1}^{{n_{D} }} {\mathbf{BV}}^{{{\mathbf{D}}_{{\mathbf{k}}} }} = \frac{1}{2} \cdot {\mathbf{BV}}^{{\mathbf{Q}}} + \overline{{{\mathbf{BV}}^{{{\mathbf{Ds}}}} }} ,$$

where $\overline{{{\mathbf{BV}}^{{{\mathbf{Ds}}}} }}$ is the mean ${\mathbf{BV}}$ of drones having mated with queen Q.

At the scale of the diploid parents, Eq. (4a) can be reformulated using Eq. (2) as:

4b $$\overline{{{\mathbf{BV}}^{{\mathbf{W}}} }} = \frac{1}{2}\left( {{\mathbf{BV}}^{{\mathbf{Q}}} + {\mathbf{BV}}^{{{\mathbf{DPQ}}}} } \right) + \frac{1}{{n_{D} }}\mathop \sum \limits_{k = 1}^{{n_{D} }} {\varvec{\upvarphi }}^{{{\mathbf{DPQ}}_{{\mathbf{k}}} }} .$$

Thus, the expectation of a colony performance can be written from Eqs. (1) and (4a) as:

5a $$E\left( P \right) = BV_{mat}^{Q} + \frac{1}{2}BV_{dir}^{Q} + \overline{{BV_{dir}^{{{\text{Ds}}}} }} ,$$

where $Q$ is the queen of the colony and $\overline{{BV_{dir}^{{{\text{Ds}}}} }}$ is the mean direct breeding value of drones that mated with this queen $Q$.

Again, this equation can be written considering only diploid parents as:

5b $$E\left( P \right) = BV_{mat}^{Q} + \frac{1}{2}\left( {BV_{dir}^{{\text{Q}}} + BV_{dir}^{{{\text{DPQ}}}} } \right).$$

Note that in Eq. (5b), contributions from Mendelian sampling terms do not appear since their expectation is zero.

In the base population (t = 0) and initial population (t = 1), queens and drones are unrelated and queens are mated to drones that are expected to come from different unrelated and non-inbred DPQ, since they are supposed to come from numerous colonies and various apiaries present in the surrounding locations. With these assumptions, the breeding values of the base drones (t from 0 to 3) follow a bivariate normal distribution with expectation 0 and variance–covariance matrix $\frac{1}{2} \cdot {{\varvec{\Sigma}}}_{{{\varvec{\tt {BV}}}}}^{{\mathbf{2}}}$, their genetic variance being half that of diploid queens: ${\mathbf{BV}}^{{{\mathbf{D}}_{{[ {\mathbf{t}} ]}} }} \sim N\left( {{\mathbf{0}},{ }\frac{1}{2} \cdot {{\varvec{\Sigma}}}_{{{\varvec{\tt {BV}}}}}^{{\mathbf{2}}} } \right)$.

The phenotypic variance of the colonies in the initial population ($V\left( {P_{[ 1 ]} } \right)$), which is marked by a subscript [1] for colonies bred by queens from the base population that are marked by a subscript [0], as developed from Eqs. (1) and (4a) depends on the number of drones $n_{D}$ as:

6 $$\begin{aligned} V\left( {P_{[1]} } \right) & = V\left( {BV_{mat}^{{Q_{[0]} }} } \right) + \frac{1}{4}V\left( {BV_{dir}^{{Q_{[ 0]} }} } \right) \\ & \quad + \frac{1}{{n_{D} }}V\left( {BV_{dir}^{{D_{[0]} }} } \right) + Cov\left( {BV_{mat}^{{Q_{[0]} }} , BV_{dir}^{{Q_{[0]} }} } \right) + V\left( e \right). \\ \end{aligned}$$

Using the predefined notations for (co)variances in the base population, Eq. (6) leads to:

7 $$\begin{aligned} E\left( {V\left( {P_{[1]} } \right)} \right) & = \sigma_{P}^{2} \\ & = \sigma_{{BV_{mat} }}^{2} + \frac{{\left( {n_{D} + 2} \right)}}{{4 \cdot n_{D} }}\sigma_{{BV_{dir} }}^{2} + \sigma_{{BV_{mat} , BV_{dir} }} + \sigma_{e}^{2} . \\ \end{aligned}$$

Although Eqs. (5a), (6) and (7) are not used in the simulations since we only derived empirical means and variances from the simulations, they are useful to understand the importance of the level of polyandry ($n_{D}$) in our results. In addition, it helps to derive the expected heritability values for direct ($h_{dir}^{2}$) and maternal ($h_{mat}^{2}$) genetic effects in the base population that depend on $n_{D}$ (see Table 1). These heritability values are calculated as the ratio between $\sigma_{{BV_{dir} }}^{2}$ or $\sigma_{{BV_{mat} }}^{2}$ and the phenotypic variance (see [30] for other measures of heritability that are relevant for response to selection in the case of honeybee breeding).

**Table 1:** Genetic and phenotypic variances, correlations between direct and maternal effects, and heritabilities for direct and maternal effects for the four parameter setups (1, 2, 3, and 4)

	Parameter setup			
	1	2	3	4
$\sigma_{{BV_{dir} }}^{2}$	10	20	10	20
$\sigma_{{BV_{mat} }}^{2}$	10	10	10	10
$r_{{BV_{dir} , BV_{mat} }}$	0	0	− 0.50	− 0.50
Monoandrous mating (queen mated with a single drone)				
$\sigma_{P}^{2}$	47.50	55.00	42.5	47.93
$h_{dir}^{2}$	0.21	0.36	0.24	0.42
$h_{mat}^{2}$	0.21	0.18	0.24	0.21
Polyandrous mating (queen mated with 8 drones)				
$\sigma_{P}^{2}$	43.13	46.25	38.13	39.18
$h_{dir}^{2}$	0.23	0.43	0.26	0.51
$h_{mat}^{2}$	0.23	0.22	0.26	0.26

$\sigma_{{BV_{dir} }}^{2}$, $\sigma_{{BV_{mat} }}^{2}$ and $\sigma_{P}^{2}$ represent, respectively, the variances ($\sigma^{2}$) of the queens’ direct ($dir$) and maternal ($mat$) breeding values ($BV$) and of the colony’s performance ($P$) in the base population; $r_{{BV_{dir} , BV_{mat} }}$ is the direct-maternal genetic correlation; $\sigma_{P}^{2}$ is the phenotypic variance in the initial population. $h_{dir}^{2}$ and $h_{mat}^{2}$ are the heritabilities for direct ($dir$) and maternal ($mat$) effects, respectively

Through simulation, we explored four parameter setups that differed in terms of (co)variance components for direct and maternal genetic effects. In these four setups, $\sigma_{{BV_{mat} }}^{2}$ remained constant, whereas $\sigma_{{BV_{dir} }}^{2}$ was either equal or twice as high as $\sigma_{{BV_{mat} }}^{2}$. In addition, the genetic correlation between direct and maternal effects ($r_{{BV_{dir} , BV_{mat} }}$) was assumed to be either zero or negative (− 0.50) in the base population.

All these values are in the range of estimates for various honeybee traits and datasets [26, 31–33].

### Haplodiploid relationship matrix

Theoretically, haplodiploid reproduction systems operate under the same genetic principles as the X-linked genes in diploid systems under the assumption of no crossover between the X and Y chromosomes [34]. Therefore, we implemented Fernando and Grossman’s [29] algorithm for the sex chromosomes to generate the relationship matrix (${\mathbf{A}}$) for all individuals in the population (see Additional file 1). Since matrix ${\mathbf{A}}$ was used in our simulation only to compute inbreeding coefficients, elements comprising information about individuals older than 3 generations were truncated each year to maintain a constant matrix size while still holding all relevant information for the inbreeding coefficients of newly born queens. This reduced greatly the RAM and running time necessary for simulations, especially for long-term runs, in which running time increased linearly with the number of generations instead of a merely quadratic relation. The simulation script was entirely written de novo. The language used for programming was R [35]. More details on the packages used are given in Additional file 1.

### Scenarios

We simulated 23 years of selection, which included three years to build up the initial breeding population from a wild base population followed by 20 years of selection in a closed population. Eighteen distinct simulation scenarios were investigated: i.e. each of the four parameter setups described in Table 1 was used in two breeding strategies, either mass selection (M) or within-maternal line selection (L), and with either monoandrous mating (1 drone per queen) or polyandrous mating (8 drones per queen for all scenarios and an additional 16 drones per queen for setup 1 only). Each scenario was independently replicated 160 times for each initial parameter setup to estimate sampling means and variances.

### Breeding population summary statistics

Several summary statistics that describe the evolution of the breeding population were calculated:

- the arithmetic mean and variance of the performance (P) of all the queen colonies for each year, and their annual means over the 160 replication runs,
- the arithmetic mean of the inbreeding (*F*) of all the queens for each year, and their annual means over the 160 replication runs,
- and the arithmetic means and variances of the direct and maternal breeding value ($BV_{{{\text{dir}}}}^{{\text{Q}}}$ and $BV_{{{\text{mat}}}}^{{\text{Q}}}$) of all the queens for each year, and their annual means over the 160 replication runs.

### Simulation of the breeding population

#### Demographic structure of the population

At the beginning of 2010, the French association of royal jelly producers (hereafter named GPGR) implemented a breeding scheme at a national scale to improve a honeybee population for traits of interest, among which quantity of royal jelly harvested during the production season, feed autonomy and gentleness of the colony.

**Fig. 2:**
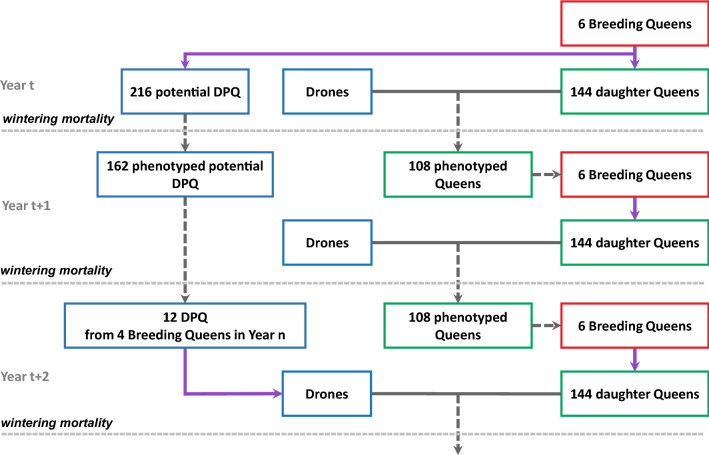
Demographic diagram of the breeding population over three successive years. The breeding scheme shown here considers a small breeding population with six breeding queens producing daughter queens each year. Drones come from drone-producing queens (DPQ) that are the best phenotyped potential DPQ who survive the two winter periods (the 1st and 2nd winters in years t and t + 1 result in the random loss of 25 and 33% of all DPQ entering winter, respectively). Blue, green and red boxes refer to sires, potential breeding queens and breeding queens, respectively. Purple and solid grey arrows indicate genetic inheritance and mating, respectively. Dotted grey arrows refer to survival or selection events from one year to another

The generation interval is only 1 year on the dam selection path (Fig. 2). Because each newborn queen is mated to several drones produced by a DPQ descending from breeding queens selected 2 years before, the generation interval is 2 years on the sire’s selection path. This longer generation interval permits a full year of performance phenotyping of the DPQ and corresponds to a progeny test of the breeding queens. Each breeding queen produces 24 virgin queens and 36 potential DPQ each year (in accordance with the GPGR breeding plan). A first winter mortality event (before phenotyping) eliminates randomly 25% of all queen colonies, leaving 108 for phenotyping, and 25% of all potential DPQ colonies. A second winter mortality event occurs (after phenotyping) at a higher rate of 33%, leading to a small proportion (108 out of the initial 216) of surviving potential 2-year old DPQ, as observed in the GPGR breeding population. While the total size of the population remains fixed over generations, the mortality events imbalance randomly each year the number of descendants per breeding queen.

#### Selection of breeding queens

Each year, six breeding queens are chosen by selecting the colonies with the best performance among 108 phenotyped queens produced by the breeding queens of the previous year. Two alternative strategies of selection are considered:

i. a full mass selection strategy (M), which is carried out among the whole population of queens, without considering the maternal pedigree of the selection candidates;
ii. and a within-maternal line selection strategy of the best candidates (L), in which all the maternal lines of the queens are conserved from one generation to the next; this strategy can be considered as a within-family selection, and corresponds to the strategy currently used by the breeding association.

It should be noted that the previously described random phenomenon of winter mortality events can extinguish a maternal line. When such an event occurs during simulation, one of the remaining lines is randomly chosen and split into two lines to create a new one, thus maintaining the initial number of maternal lines, in accordance with GPGR’s usual practice.

#### Selection and mating of drone-producing queens

Drone-producing queens were selected in two steps, i.e. family selection followed by within-family selection (see Additional file 1: Figure S1 for an illustration):

- Step (1): four sib groups of potential DPQ (a sib group being the progeny of the same breeding queen) were selected by choosing the four families with the best average potential DPQ colony performance out of the six families (if there was at least one surviving potential DPQ in each family; otherwise the next best ranking family with at least one survivor was selected). This first step corresponded to a 66% selection rate when all families survived through the winter period.
- Step (2): in each selected sib group, the three best performing potential DPQ were then chosen to become the actual DPQ, within the limit of the number of potential DPQ surviving after the second winter period. If only one or two sisters of the potential DPQ survived in a selected family, only these queens were used to produce all the drones expected from this sib group, in accordance with the GPGR’s common practice.

It is only in the 4th year of the breeding plan that the DPQ were selected from the breeding population to produce drones. In previous years, drones came from unselected colonies present in the environment. A DPQ was randomly chosen to produce 1, 8 or 16 drones that mated with each virgin queen. Each DPQ sib group participated equally to the drone pool.

## Results

Depending on the parameter setups (see Table 1), Tables 2 and 3 show the phenotypic, genetic and inbreeding levels after 20 years of selection in the closed populations simulated for 18 scenarios that represent combinations of four initial genetic parameters with two selection strategies and three mating levels (monoandry and polyandry with 8 or 16 drones). To facilitate the comparisons across scenarios, the performance was standardized by the initial phenotypic standard deviation and the breeding values by their initial genetic standard deviation.

**Table 2:** Phenotypic and genetic evolution of the population over 20 years for the parameter setups without an initial direct–maternal correlation

Selection strategy	Setup	$n_{D}$	*F* (%)	$\frac{{P_{{[{24}]}} }}{{\sigma_{P} }}$	$\frac{{BV_{dir}^{{Q_{{[ {23} ]}} }} }}{{\sigma_{{BV_{dir} }} }}$	$\frac{{BV_{mat}^{{Q_{{[ {23} ]}} }} }}{{\sigma_{{BV_{mat} }} }}$	$V\left( {P_{{[ {24} ]}} } \right)$	$V\left( {BV_{dir}^{{Q_{{[ {23} ]}} }} } \right)$	$V\left( {BV_{mat}^{{Q_{{[ {23} ]}} }} } \right)$	$r_{{BV_{dir} , BV_{{mat_{{[ {23} ]}} }} }}$
L	1	1	25.68 (2.56)	4.24 (0.49)	5.92 (0.81)	3.45 (0.72)	40.09 (6.29)	5.47 (2.23)	5.88 (2.67)	− 0.089 (0.275)
		8	19.49 (2.19)	4.63 (0.39)	4.62 (0.63)	5.10 (0.64)	40.88 (5.93)	6.68 (2.20)	6.66 (2.15)	− 0.030 (0.197)
		16	18.57 (2.14)	4.82 (0.42)	4.60 (0.60)	5.43 (0.63)	39.31 (6.19)	6.89 (1.91)	6.38 (2.02)	− 0.074 (0.203)
	2	1	27.15 (3.36)	5.81 (0.54)	7.74 (0.80)	3.11 (0.79)	43.72 (7.50)	10.04 (4.41)	5.73 (2.58)	− 0.022 (0.265)
		8	20.23 (2.18)	6.11 (0.48)	6.07 (0.65)	4.79 (0.67)	42.64 (6.54)	12.50 (3.64)	6.48 (2.14)	− 0.036 (0.206)
M	1	1	38.72 (4.97)	5.27 (0.50)	6.20 (0.90)	5.27 (0.87)	38.00 (5.57)	4.40 (1.87)	4.55 (2.22)	− 0.062 (0.283)
		8	26.58 (3.63)	5.53 (0.49)	5.11 (0.70)	6.47 (0.75)	37.72 (5.27)	5.92 (1.89)	5.76 (1.83)	− 0.060 (0.185)
		16	25.51 (3.23)	5.56 (0.44)	4.93 (0.77)	6.61 (0.67)	38.51 (5.00)	5.95 (7.89)	5.98 (1.60)	− 0.054 (0.182)
	2	1	38.94 (5.01)	6.76 (0.55)	7.96 (0.86)	4.88 (0.79)	40.82 (6.24)	7.98 (3.58)	4.47 (1.83)	− 0.085 (0.289)
		8	27.30 (4.05)	7.11 (0.53)	6.68 (0.73)	6.12 (0.68)	41.08 (6.06)	11.01 (3.17)	5.84 (1.98)	− 0.064 (0.205)

Values in brackets represent sampling standard deviations over 160 replicates

Parameter setups 1 and 2 are fully described in Table 1. In setup 1, direct ($dir$) and maternal ($mat$) genetic variances in the base population are equal ($\sigma_{{BV_{dir} }}^{2} = \sigma_{{BV_{mat} }}^{2}$), while in parameter setup 2, $\sigma_{{BV_{dir} }}^{2} = 2 \cdot \sigma_{{BV_{mat} }}^{2}$. In both setups, the direct-maternal genetic correlation in the base population is null.

L is within-maternal-line selection; M is mass selection; $n_{D}$ is number of drones mating each queen; *F* is the inbreeding coefficient; $\frac{{P_{{[ {24} ]}} }}{{\sigma_{P} }}$ is the standardized performance of colonies in year 24; $\frac{{BV_{dir}^{{Q_{{[ {23} ]}} }} }}{{\sigma_{{BV_{dir} }} }}$ and $\frac{{BV_{mat}^{{Q_{{[ {23}]}} }} }}{{\sigma_{{BV_{mat} }} }}$ are the direct and maternal standardized breeding values of queens born in year 23, respectively; $V\left( {P_{{[ {24} ]}} } \right)$ is the phenotypic variance of colonies performing in year 24; $V\left( {BV_{dir}^{{Q_{23} }} } \right)$ and $V\left( {BV_{mat}^{{Q_{23} }} } \right)$ are the direct and maternal genetic variances of queens born in year 23; $r_{{BV_{dir} , BV_{{mat_{{[ {23}]}} }} }}$ is the genetic correlation between direct and maternal effects from queens born in year 23.

**Table 3:** Phenotypic and genetic evolution of the population over 20 years for the parameter setups with an initial negative direct-maternal correlation

Selection strategy	Setup	$n_{D}$	*F* (%)	$\frac{{P_{{[ {24} ]}} }}{{\sigma_{P} }}$	$\frac{{BV_{dir}^{{Q_{{[ {23} ]}} }} }}{{\sigma_{{BV_{dir} }} }}$	$\frac{{BV_{mat}^{{Q_{{[ {23} ]}} }} }}{{\sigma_{{BV_{mat} }} }}$	$V\left( {P_{{[ {24} ]}} } \right)$	$V\left( {BV_{dir}^{{Q_{{[ {23} ]}} }} } \right)$	$V\left( {BV_{mat}^{{Q_{{[ {23} ]}} }} } \right)$	$r_{{BV_{dir} , BV_{{mat_{{[ {23} ]}} }} }}$
L	3	1	26.11 (3.18)	2.42 (0.39)	4.41 (0.79)	0.75 (0.74)	37.35 (6.15)	5.67 (3.06)	6.16 (3.20)	− 0.491 (0.240)
		8	18.83 (2.38)	2.69 (0.35)	2.11 (0.66)	3.26 (0.64)	37.01 (5.64)	6.99 (2.43)	6.97 (2.51)	− 0.525 (0.148)
	4	1	26.47 (2.89)	3.79 (0.44)	6.31 (0.81)	− 0.35 (0.79)	40.93 (6.09)	11.11 (4.63)	5.57 (2.44)	− 0.461 (0.219)
		8	20.45 (2.56)	3.85 (0.42)	3.92 (0.68)	2.18 (0.68)	38.69 (5.22)	13.38 (3.93)	6.87 (2.11)	− 0.526 (0.155)
M	3	1	35.42 (4.93)	3.07 (0.41)	4.02 (0.98)	2.43 (0.98)	36.08 (5.00)	5.03 (2.59)	5.15 (2.67)	− 0.528 (0.217)
		8	24.41 (3.63)	3.29 (0.37)	2.02 (0.75)	4.51 (0.75)	35.89 (4.68)	6.10 (1.99)	6.37 (1.86)	− 0.518 (0.131)
	4	1	36.90 (4.17)	4.44 (0.48)	6.45 (0.83)	0.95 (0.88)	39.43 (5.90)	9.22 (4.10)	4.88 (2.37)	− 0.503 (0.232)
		8	25.41 (3.47)	4.44 (0.46)	4.05 (0.71)	3.14 (0.70)	37.68 (5.15)	12.52 (4.04)	6.13 (2.05)	− 0.522 (0.141)

Values in brackets represent sampling standard deviations over 160 replicates

Parameter setups 3 and 4 are fully described in Table 1. In setup 3, direct ($dir$) and maternal ($mat$) genetic variances in the base population are equal, while in parameter setup 4, $\sigma_{{BV_{dir} }}^{2} = 2 \cdot \sigma_{{BV_{mat} }}^{2}$. In both setups, the direct-maternal genetic correlation in the base population equals − 0.5.

L is within-maternal-line selection; M is mass selection; $n_{D}$ is the number of drones mating each queen; *F* is the inbreeding coefficient; $\frac{{P_{{\left[ {24} \right]}} }}{{\sigma_{P} }}$ is the standardized performance of colonies in year 24; $\frac{{BV_{dir}^{{Q_{{\left[ {23} \right]}} }} }}{{\sigma_{{BV_{dir} }} }}$ and $\frac{{BV_{mat}^{{Q_{{\left[ {23} \right]}} }} }}{{\sigma_{{BV_{mat} }} }}$ are the direct and maternal standardized breeding values of queens born in year 23, respectively; $V\left( {P_{{\left[ {24} \right]}} } \right)$ is the phenotypic variance of colonies performing in year 24; $V\left( {BV_{dir}^{{Q_{23} }} } \right)$ and $V\left( {BV_{mat}^{{Q_{23} }} } \right)$ are the direct and maternal genetic variances of queens born in year 23; $r_{{BV_{dir} , BV_{{mat_{{\left[ {23} \right]}} }} }}$ is the genetic correlation between direct and maternal effects from queens born in year 23.

### Impacts of the selection strategy and polyandry on performance and inbreeding levels after 20 years of selection

The standardized performance after 20 years of selection varied strongly depending on the level of polyandry considered, the selection strategy and the initial parameter setup. For all scenarios, we observed strong increases in performance and inbreeding levels after 20 years of selection in closed populations. Regarding performance increases, differences across parameter setups were substantial. For a given polyandry level and selection strategy, the best-performing setup (setup 2) was that with an initial direct genetic variance twice as high as the maternal genetic variance. It resulted in a mean performance (Table 2) more than 1.2 times higher than the worst performing setup (setup 3) with the same initial variances for direct and maternal effects and a negative correlation between those effects (Table 3). Regarding inbreeding levels, the highest increases in inbreeding were observed under mass selection (M) with monoandry ($n_{D}$ = 1) for all parameter setups. For a given selection and mating strategy, the increases in inbreeding were very similar across parameter setups.

Under setup 1, the lowest performance means were observed under within-family selection with monoandry ($n_{D}$ = 1). Moving from a monoandrous to polyandrous mating system increased by 9% the mean performance reached after 20 years of within-family selection. With setup 1, gains in performance were more homogenous under mass selection, polyandry yielding only a 5% increase compared to monoandry. At a fixed level of polyandry, mass selection resulted on average in a 20% higher performance than within-family selection.

Similar trends were observed for all the other parameter setups. Mass selection enabled greater (from + 15 to + 27%) gains in performance than within-family selection. The differences in final performance between mass and within-family selection were greater in the scenarios with monoandry than in those with polyandry and for setups with the same initial direct and maternal variances. Polyandrous mating generally induced a better gain in performance (from 0 to + 11%) than monoandrous mating, especially for parameter setups with the same initial direct and maternal variances. On the opposite, in the two alternative setups, and especially for setup 4, polyandrous mating brought practically no gain compared to monoandrous mating.

**Fig. 3:**
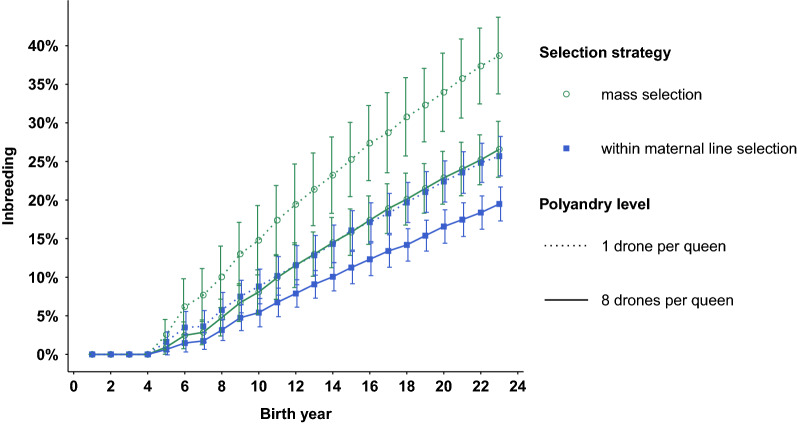
Evolution of the average inbreeding of queens under mass or within-maternal line selection with a monoandrous or polyandrous mating system. Inbreeding increases almost linearly from year 7 (after 4 years of closed-population breeding) onwards, with an annual increase of 1.1% in the within-maternal line selection scenario with polyandrous mating to 1.9% in the mass selection scenario with monoandrous mating, reaching 10% in years 14 and 8, respectively. The evolution of inbreeding levels under within-maternal line selection with monoandry and under mass selection with polyandry is very similar. In these two scenarios, inbreeding increases annually by 1.5% after year 7, reaching 10% in year 11. Bars represent 2 times the sampling standard deviation over the 160 simulation replicates

The evolution of the inbreeding levels of the queens over years is shown for the four scenarios with parameter setup 1 in Fig. 3. In all four scenarios, inbreeding increased almost linearly from year 7 (after 4 years of closed-population breeding) onwards, at rates ranging from 1.1% per year under within-family selection with polyandry to 1.9% per year under mass selection with monoandry. Thus, the average inbreeding level of the queens reached 10% between years 8 and 14. Interestingly, we observed a very similar evolution of inbreeding levels under within-family selection with monoandry than under mass selection with polyandry. In these latter two scenarios, the average inbreeding level of queens reached 10% in year 11. Similar trends were observed for all parameter setups.

For setup 1, regardless of the polyandry level, inbreeding levels after 20 years of selection were significantly higher in the scenarios with mass selection (from 26 to 39%) than in those with within-family selection (from 19 to 26%). Compared to the scenarios with mass selection, in those with within-family selection the sampling standard deviations over replicates of the inbreeding level were also drastically reduced (about − 40%). While there was only a small difference (1%) observed in the final inbreeding levels under the two polyandrous mating scenarios (with $n_{D}$ = 8 and $n_{D}$ = 16) with the same selection strategy (Table 2), monoandrous mating induced strong increases in inbreeding levels compared to polyandrous mating (+ 32% and + 46% after 20 years of within-family and mass selection, respectively, taking $n_{D}$ = 8 as the polyandrous reference).

These observations were similar for all the other parameter setups, in which mass selection resulted in a 24 to 43% increase of inbreeding levels compared to within-family selection, with the largest differences observed for the scenarios with monoandry and uncorrelated direct and maternal genetic effects in the base population. Again, for all parameter setups, we observed a similar evolution of inbreeding levels under within-family selection with monoandry than under mass selection with polyandry.

### Impacts of the selection strategy and polyandry on standardized direct and maternal genetic gains

**Fig. 4:**
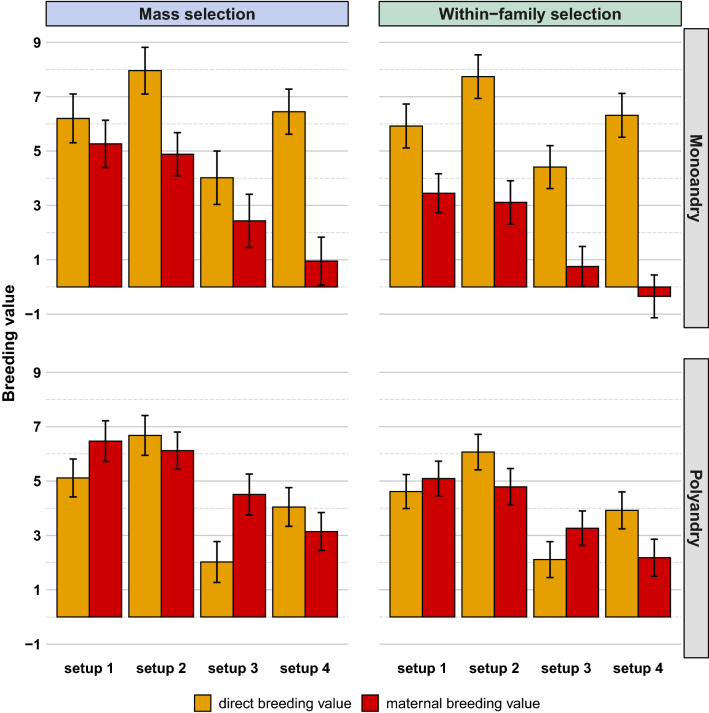
Direct and maternal average standardized breeding values of queens after 20 years of selection for all simulated scenarios. For each parameter setup, polyandry level and selection strategy, brown and red bars represent the direct and maternal average breeding values of queens after 20 years of selection, respectively. Deviation bars represent two times the sampling standard deviation over the 160 simulation replicates. For the setup parameters, see Table 1

Regarding the genetic factors that explain colony performance, we observed contrasted gains in direct *versus* maternal genetic effects (Fig. 4) even for the parameter setups that considered the same initial genetic variances for both effects in the base population (setups 1 and 3).

Under setup 1, all scenarios showed strong increases in both direct and maternal breeding values of the queens after 20 years of selection in a closed population (Fig. 4). Monoandrous mating induced higher genetic gains for direct effects than for maternal effects regardless of the selection strategy, while the opposite result was observed with polyandrous mating. The two ‘polyandry’ scenarios led to very similar results for a given selection strategy (Table 2). The relative genetic gains for maternal versus direct effects were contrasted between scenarios with monoandry versus polyandry, and these differences varied between selection strategies. The average $BV_{dir}^{Q}$ were about 20% higher in the scenarios with monoandry than in the corresponding ones with polyandry (Fig. 4). On the opposite, the average $BV_{mat}^{Q}$ were always lower in the scenarios with monoandry (about − 38% and − 23% for within-family and mass selection, respectively) (Fig. 4). Mass selection induced the highest direct genetic gains (+ 5% in the scenarios with monoandry and + 11% in those with polyandry) compared to within-family selection, but induced even much more maternal genetic gains (+ 53% in the scenarios with monoandry and + 27% in those with polyandry). The largest difference between direct and maternal genetic gains was observed under within-family selection with monoandry. In this scenario, the average $BV_{dir}^{Q}$ was 72% higher than the average $BV_{mat}^{Q}$ after 20 years of selection. Under within-family selection with polyandry, the direct and maternal genetic gains were closer, with the average $BV_{dir}^{Q}$ being only 9% lower than $BV_{mat}^{Q}$ after 20 years of selection. Compared to the results observed with within-family selection, the difference between direct and maternal genetic gains was reduced under mass selection with monoandry (average $BV_{dir}^{Q}$ being only 18% higher $BV_{mat}^{Q}$), and slightly increased under mass selection with polyandry (the average $BV_{dir}^{Q}$ being 21% lower than $BV_{mat}^{Q}$).

Although the absolute values of the direct and maternal genetic gains strongly varied depending on the initial parameter setups, direct genetic gains were always higher than maternal genetic gains in all the scenarios with monoandry, but in the scenarios with polyandry this was the case only when the direct genetic variance was twice the maternal genetic variance (Fig. 4). This doubling of the direct genetic variance permitted an increased direct genetic gain over its maternal counterpart, even after standardizing breeding values by their initial standard deviations to account for the different initial variances (Table 2).

Setting an initial negative correlation between direct and maternal effects (setups 3 and 4) reduced both genetic gains significantly and exacerbated differences in their relative progress. In setup 4, in which the unbalanced genetic variances and the negative correlation between direct and maternal effects were combined, we even observed a slight negative genetic trend for maternal effects under within-family selection with monoandry. In setups 3 and 4, mass selection induced a similar (setup 4) or even a little lower (setup 3) genetic gain on direct effects than within-family selection. Nevertheless, for these setups, mass selection permitted higher gains on maternal effects than within-family selection.

### Impacts of the selection strategy and polyandry on the genetic parameters

**Fig. 5:**
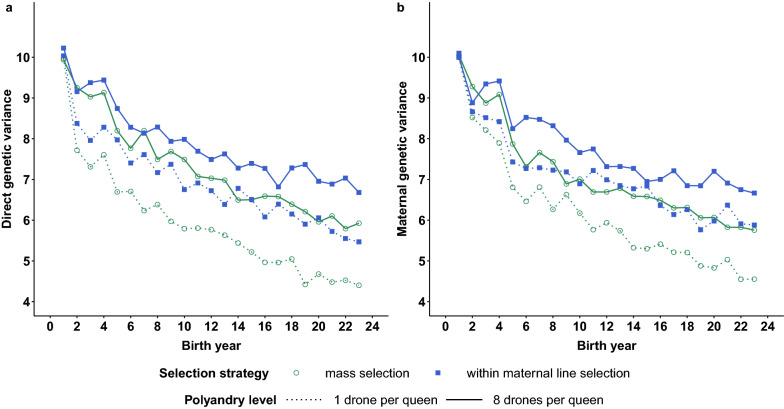
Evolution of the average direct and maternal genetic parameters of queens under mass or within-maternal line selection with a monoandrous or polyandrous mating system. The evolution of the mean direct (**a**) genetic variance for the four scenarios of setup 1 is very similar to that of the mean maternal (**b**) genetic variance. Two significant decreases in variance take place between years 1 and 2 (first selection) and between years 4 and 5 (first selection in the closed population). Until year 4, the loss in direct and maternal genetic variance is essentially due to the Bulmer effect, whereas loss continues subsequently as inbreeding increases. Within-maternal line selection with polyandrous mating maintains the highest genetic variance, whereas mass selection with monoandrous mating induces the most severe losses. The within-maternal line selection with monoandry and mass selection with polyandry scenarios produced similar intermediate losses

Regarding the evolution of the genetic variances (Table 2) in setup 1, they decreased more strongly with monoandry (~ − 44% under within-family selection, for example) than with polyandry (~ − 34% under within-family selection, for example). These decreases were more important under mass selection (~ − 55% with monoandry) than under within-family selection because of the stronger reductions in the genetic variances associated with the higher selection intensity. This so-called Bulmer effect induced rapid decreases of both the direct and maternal genetic variances under mass selection with monoandry within the first five years of selection (Fig. 5). A first steep decrease in variance was observed between years 1 and 2 due to the first step of queen selection. A second strong decrease in variance occurred between years 4 and 5 and corresponded to the first generation of selection in a closed population, i.e. on queens mated to drones bred by the first DPQ chosen within the breeding population. In the longer term, the decrease in variance is due to the increase in inbreeding level (Fig. 3) with a stronger reduction in genetic variances in the scenarios with monoandry associated with higher inbreeding levels than in scenarios with polyandry. Regardless of the selection strategy and polyandry level considered, very similar decreases were observed for both direct and maternal genetic variances although the corresponding genetic gains could differ quite notably (Fig. 5).

For all the parameter setups, we observed that additional decreases in the direct and maternal variances of about 21 and 18%, respectively, relative to their values in the base population, occurred under mass selection with monoandry compared to the loss under within-family selection with polyandry.

The evolution of the direct-maternal correlations after 20 years of selection shows that the changes were on average modest, and mostly negative (Table 2). However, the high sampling variances derived for the estimate indicate that the correlation between direct and maternal effects was quite variable across years and replicates. Stronger decreases of the correlation were associated with an initial null correlation and the mass selection scenarios.

## Discussion

After 20 years of selection in a closed population and regardless of the initial parameter setup that was simulated, the breeding scheme based on mass selection with polyandrous mating produced the greatest improvement in performance. Considering a monoandrous mating system did not induce any further gain, but led to a very high increase in inbreeding compared to the polyandrous mating system.

### Genetic trends on direct and maternal effects

Regarding maternal genetic effects, regardless of the mating system, the performance of a colony depends only on the maternal breeding value of its unique queen. Compared to polyandrous mating, monoandrous mating allows a higher male selection intensity and thus favors a selection response for direct genetic effects that are expressed in both the male and female paths. Regarding the direct genetic effects, the performance of a colony depends on the direct breeding value of the queen and, depending on the mating system, either on the direct breeding value of a single drone or on the average of the direct breeding values of $n_{D}$ drones. Assuming that the drones come from a single DPQ (as is the case after year 3), the phenotypic variance can be approximated in year t under the (false) assumption of unrelated breeding queens and DPQ as:

8 $$\begin{aligned} V\left( {P_{[ t ]} } \right) & = V\left( {BV_{mat}^{{Q_{{[ {t - 1}]}} }} } \right) + \frac{{\left( {n_{D} + 2} \right)}}{{4 \cdot n_{D} }}V\left( {BV_{dir}^{{Q_{{[ {t - 1} ]}} }} } \right) \\ & \quad + Cov\left( {BV_{mat}^{{Q_{{[ {t - 1} ]}} }} , BV_{dir}^{{Q_{{[ {t - 1} ]}} }} } \right) + V\left( e \right). \\ \end{aligned}$$

As also shown by Eq. (7) for the initial population, the weighting of direct and maternal effects in the phenotypic variance varies with the polyandry level ($n_{D}$). Regardless of the level of polyandry, the phenotypic variance depends more on the maternal genetic variance than on the direct genetic variance. However, in the scenarios with monoandrous mating, the weighting coefficient of the direct effects in Eq. (8) is equal to ¾ while it tends to be equal to ¼ when $n_{D}$ becomes large. This partly explains why a stronger response to selection was observed for direct effects in the scenarios with monoandry than in those with polyandry.

For a long time, evolution models have explicitly modelled and considered the specific genetic and evolutionary features of social traits in social insects. These models enabled us to compare our results obtained under artificial selection to those derived under natural selection. Both individual and colony phenotypes of social insects are influenced by genes expressed zygotically (direct effects) as well as by genes expressed in social partners (indirect effects such as maternal effects or sib effects). Social insect adult workers are expected to simultaneously affect the fitness of their mother, through offspring effects, and the fitness of their younger reproductive siblings (new males and queens), through sib effects [36]. Sib effects are considered under kin selection models, which aim at taking “offspring control” into account in the case of the evolution of eusociality, because whether an offspring helps raise its sibs depends directly on its own genotype [37]. Maternal effects are considered in parental manipulation models in which the genes involved are located in the maternal genome. Because of these differences in the location of genes underlying the behaviors, the cost to benefit ratio that is necessary for alleles to spread by parental manipulation (i.e. maternal effects) is often half that of kin selection and thus parental manipulation alleles spread more easily [37]. This general result corresponds to a certain extent to our weighting of maternal effects *vs* direct effects in the phenotypic variance under selection (Eq. 8). Using a maternal effects model, Wade [38] showed that alleles affecting maternal effects fix more easily in haplodiploids than in diplodiploids. In addition, Wade showed that multiple mating does not restrict the evolution of genes with maternal effects as it does for kin selection genes. This last observation confirms our results that polyandry tends to favor genetic progress on maternal effects compared to monoandry.

Linksvayer and Wade [36] modeled how the fitness of new reproducers depends directly on genes expressed in these individuals, as well as indirectly on genes expressed in the mother (i.e., the queen) and in their siblings (i.e., workers). Linksvayer and Wade [36, 39] showed that under natural selection, fitness traits that depend on worker genes have a reduced selection potential compared to fitness traits that depend on queen genes.

Under artificial selection, Plate et al. [23] modelled larger breeding schemes than those in our study, with only polyandrous mating and a weaker mass selection intensity on the female path. They also simulated a breeding strategy with longer generation intervals (2 years on the dam path and three years on the sire path) and a different mating strategy in which sister queens were all mated to one group of sister DPQ in which each DPQ participated randomly to the drone pool. Assuming that the initial direct genetic variance was twice the maternal genetic variance and that the correlation between the two effects was negative, these authors observed that the direct effects improved more than the maternal effects under BLUP selection based on the worker groups' estimated breeding values. However, varying the ratio between direct and maternal genetic variances (see their appendix 3), they showed that this result only holds when the genetic variance of the direct effects exceeds one and a half times the genetic variance of the maternal effects. Considering the same direct and maternal heritability, they found that the selection for maternal effects was significantly stronger than selection for direct effects, especially in the case of a strong negative correlation between these effects. Using only colony performance as selection criterion, we confirm all these previous results. In addition, we point out that the genetic trend for direct effects is favored by monoandry, and surpasses genetic gain on maternal effects when a similar genetic variation exists for both effects in the base population. This phenomenon is amplified for within-family selection compared to mass selection, or when a negative correlation exists between direct and maternal genetic effects. In the honeybee, the performances of queens (maternal effect) and workers (direct effect) are always seen together, making it difficult to correctly separate their respective effects. This could explain, in part, the frequently calculated negative estimates of the correlation between these effects. Evidence of the biological rationale of these estimates is not obvious and thus can be questioned. In the literature dedicated to estimates of genetic correlation between direct and maternal effects in terrestrial livestock, negative estimates are often derived, but should always be questioned as potential statistical artefacts [40–43]. For instance, some biological evidence has shown that the negative estimates for the genetic correlation between direct and maternal effects on weaning weight in beef cattle are unlikely to exist [44, 45]. Nevertheless, if such negative estimates are true, one plausible hypothesis may be linked to resource allocation issues. For honeybees, one can imagine the following trade-off: a queen with a high maternal breeding value will produce a large number of eggs, thus increasing the colony size and, potentially, the honey yield; but, meanwhile, the brood care quality and hence life expectancy of workers might be hampered by a too high egg laying rate and may require more resources to raise a “standard” worker bee. Thus, further simulation studies are needed to provide more insights on the reality and the impact of a negative correlation between direct and maternal effects in honeybee breeding schemes since the potential negative correlation between direct and maternal effects has long been known to complicate breeding decisions.

Concerning the evolution of direct and maternal genetic variances (Tables 2 and 3), we observed, after only 20 years of mass selection with polyandry, similar losses (− 35 to − 45%) to the losses observed by Plate et al. [23] after 100 years under an infinitesimal genetic model. Our larger losses may be explained by both a stronger selection intensity on the maternal path, and a breeding population with shorter generation intervals and a smaller size.

### Evolution of inbreeding

Monoandry increased inbreeding and consequently reduced the effective population size compared to polyandry. In our study, the only effect of inbreeding that was considered in the model was the loss of genetic variance due to less Mendelian sampling variance within families. We did not integrate in our model any inbreeding depression effect as was proposed by Moritz [20] through his regression coefficient of colony performance on inbreeding, or by Omholt and Adnoy [22] through their modelling of genetic sex determinism accounting for 15 different *csd* alleles segregating in their base population. However, our general conclusion that mass selection brought a significantly higher genetic gain (around + 20% across our scenarios) than within-family selection at the cost of a significantly higher inbreeding level (around + 35% across our scenarios) is consistent with these previous studies. Although theoretically well-founded, Moritz’ [20] approach should rely on robust estimates of inbreeding effects, that require both large datasets as well as estimates obtained for different honeybee populations with diverse origins and breeding histories and for diverse colony traits recorded across various environmental conditions [18, 19]. To our knowledge, there is only one study that has quantified inbreeding depression for several colony traits recorded by German and Austrian honeybee breeders [46]. Omholt and Adnoy [22], on their side, derived for 20 generations of selection the expected occurrence of homozygotes at the *csd* locus in the brood, corresponding to diploid males. They showed that mass selection increased the occurrence of diploid males by 25% compared to within-maternal line selection. This trend is consistent with our inbreeding trends, which were estimated to increase by 25% after 20 years (15 generations in our case) of mass *vs* within-family selection. However, the direct modelling at the *csd* locus does not appear as crucial as previously thought and the issue of diploid males could have been over-estimated. In fact, recent investigations [6] showed that the number of *csd* alleles has been largely under-estimated (from 15 previously to more than 100 alleles shared between common farmed European honeybee populations). In addition to this, and most importantly and relevant to our simulations, a high mutation and recombination rate create a large regular flow of new alleles, which counteract the occurrence of homozygotes at this locus even at high general inbreeding rates.

Using the actual size of the GPGR breeding program, we predicted that unbearably high inbreeding levels will be reached under mass selection, in particular when combined with monoandry, but also with polyandry. Inbreeding rates could probably drastically be reduced by increasing the proportion of breeding queens, which would probably only slightly decrease genetic gain, as suggested by Plate et al. [24] for small breeding populations.

To reach the highest possible genetic gains while limiting inbreeding rate in honeybee breeding populations, further developments of the simulation program should consider BLUP evaluation applied to any honeybee population structure [47, 48] associated to an optimum contribution selection strategy [49].

### Interest for monoandry in honeybee breeding programs

Monoandry is currently used by several honeybee breeding groups for specific selection objectives, such as selection for resistance to varroa. Harbo [14, 15] showed how monoandry can facilitate the discrimination of specific colony behaviors (such as varroa specific hygienic behavior), because single-drone inseminated queens produce only super-sisters with an expected relationship coefficient of 0.75 (ignoring inbreeding) and thus breed more homogeneous colonies. However, if used over several generations in a closed population, monoandry increases the inbreeding levels of queens much more than polyandry, which constitutes a strong limitation of this strategy. Another interesting strategy could be to consider monoandry not on the breeding stock, but rather on their sisters or daughters that would be phenotyped to better discriminate the genetic merit of the selection candidates. In such breeding programs, polyandrous mating for breeding queens will allow to maintain low inbreeding rates while monoandrous mating on relatives will allow a more accurate selection. Another possibility would be to use a combination of these mating strategies, starting with monoandry when the inbreeding levels are low and continuing with polyandry afterwards. Lastly, under BLUP selection, monoandry should theoretically enable a more accurate evaluation because full knowledge of the paternal pedigree is available compared to the probabilistic approach used under polyandrous mating. However, as described by Harbo [14], colonies led by single-drone inseminated queens suffer higher mortality. Their comparison with colonies led by polyandrous mated queens is not straightforward because of differences on non-genetic aspects such as lower sperm content in the spermatheca of the former queens, which were not accounted for in the simulation.

## Conclusions

Our study is the first one that compares long-term effects of monoandrous mating compared to polyandrous mating in honeybee breeding programs, the former being an emergent strategy for improving specific traits such as resistance to varroa or other traits that are important to select but difficult or expensive to phenotype. However, if used over several generations in a closed population, monoandry increases the inbreeding levels of queens much more than polyandry, which constitutes a strong limitation of this strategy in small honeybee breeding populations. Furthermore, no evidence for its potential to increase long-term performance was observed in our simulations, since the higher direct genetic gain due to monoandrous mating was counterbalanced by a lower maternal genetic improvement in all tested scenarios. From a practical perspective, we expect that high levels of polyandry and an increased number of drone-producing queens may better maintain the genetic variance as this would greatly weaken the selection intensity on the paternal path. Such a measure may decrease genetic gain in the short-term, but it may be profitable in the long-term. Increasing the number of breeding queens would be another option that could preserve the genetic gain in the short-term while maintaining the genetic variability in the long-term. If the size of the breeding population cannot be increased in any way, a last option is to open the breeding scheme to imported queens with high breeding values, thus preserving the genetic level while increasing the genetic diversity of the breeding population.

## Supplementary Information

**Additional file 1: Figure S1.** Illustration of the selection procedure of drone-producing queens. Year t: all potential drone-producing queens (DPQ) surviving the first wintering are evaluated and participate to their family mean performance. Year t + 1: The four families with the highest mean performance are selected when at least one potential DPQ had survived the second wintering. In this illustration, the high-performance scoring family 5 lost all its potential DPQ during the second wintering and thus cannot be selected. Thus, the family with the fifth ranked performance is selected. Year t + 1: For each selected family, the three sister queens with the highest own performance are selected as DPQ. If less than three sisters survived the second wintering (as for family 3 in this illustration), all surviving sisters are selected as DPQ to produce the same number of drones as expected from any DPQ family (to balance the contribution of each family to the drone pool). Green frames refer to selected families and queens and red frames refer to eliminated families.

**Additional file 2. **Relationship matrix’ algorithm. Fernando and Grossman’s (1990) algorithm for sexual chromosomes in diploid species is described. It was used to generate the relationship matrix (**A**) for all individuals in the haplodiploid bee population.

**Additional file 3. **R Packages used for programming. List of R packages used in the simulation program.

## Data Availability

The datasets and R code generated during the current study are not publicly available while the authors continue to perform additional analyses and software developments. They may be available from the corresponding author upon reasonable request.
