# Supplementary figures and images for: A simulation study of a honeybee breeding scheme accounting for polyandry, direct and maternal effects on colony performance
Source: Genet Sel Evol. 2021 Sep 8;53:71. doi: 10.1186/s12711-021-00665-8 (PMC8425095; doi:10.1186/s12711-021-00665-8)

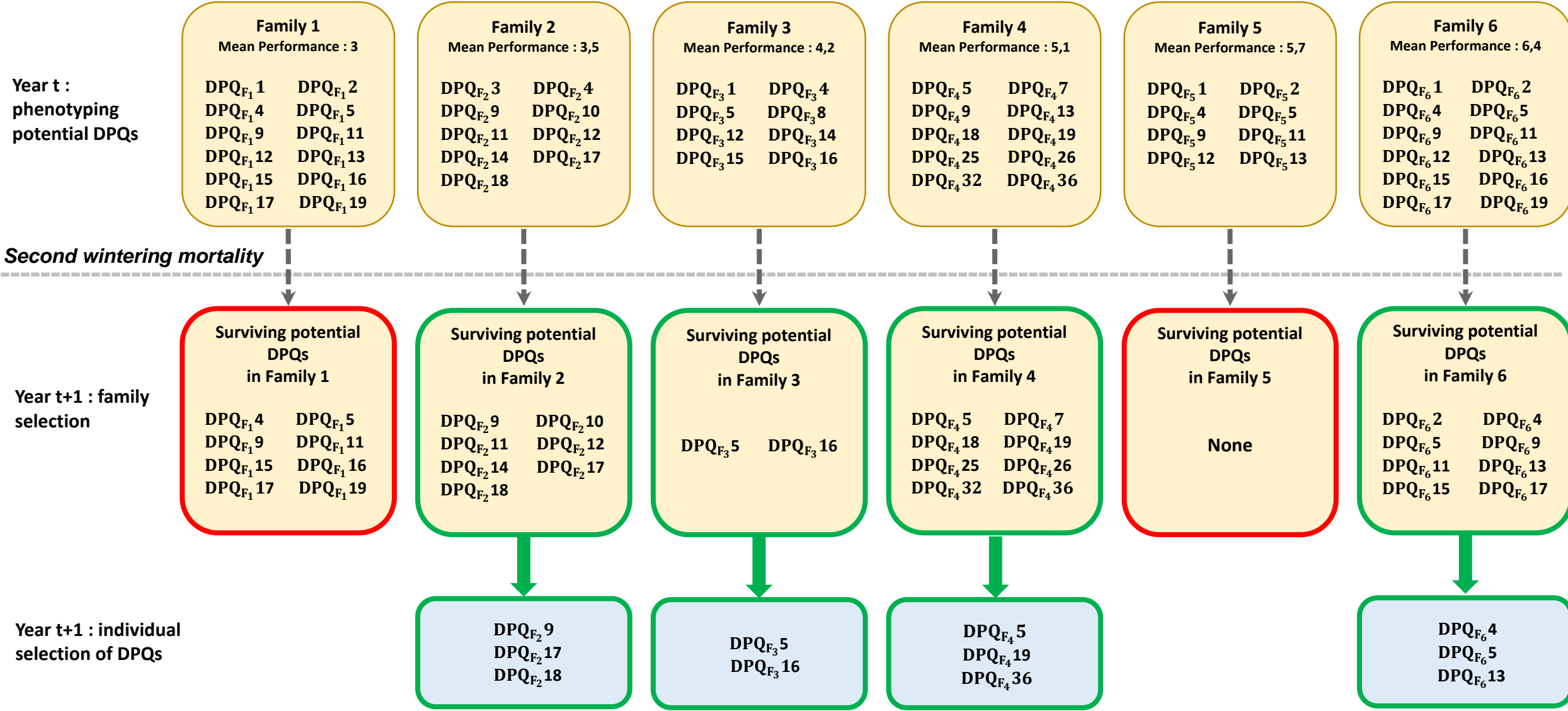

Supplement: Supplementary file 1 — Additional file 1: Figure S1. Illustration of the selection procedure of drone-producing queens. Year t: all potential drone-producing queens (DPQ) surviving the first wintering are evaluated and participate to their family mean performance. Year t + 1: The four families with the highest mean performance are selected when at least one potential DPQ had survived the second wintering. In this illustration, the high-performance scoring family 5 lost all its potential DPQ during the second wintering and thus cannot be selected. Thus, the family with the fifth ranked performance is selected. Year t + 1: For each selected family, the three sister queens with the highest own performance are selected as DPQ. If less than three sisters survived the second wintering (as for family 3 in this illustration), all surviving sisters are selected as DPQ to produce the same number of drones as expected from any DPQ family (to balance the contribution of each family to the drone pool). Green frames refer to selected families and queens and red frames refer to eliminated families. [file 12711_2021_665_MOESM1_ESM.pdf]
