# Supplementary material for: A simulation study of a honeybee breeding scheme accounting for polyandry, direct and maternal effects on colony performance
Source: Genet Sel Evol. 2021 Sep 8;53:71. doi: 10.1186/s12711-021-00665-8 (PMC8425095; doi:10.1186/s12711-021-00665-8)
Supplement: Supplementary file 2 — Additional file 2. Relationship matrix’ algorithm. Fernando and Grossman’s (1990) algorithm for sexual chromosomes in diploid species is described. It was used to generate the relationship matrix (A) for all individuals in the haplodiploid bee population. [file 12711_2021_665_MOESM2_ESM.docx]

# Additional file 2

## **Relationship matrix’ algorithm**

Fernando and Grossman’s (1990) algorithm for sexual chromosomes in diploid species is used to generate the relationship matrix (**A**) for all individuals in the haplodiploid bee population.

(1) Number individuals such that progeny follow parents.

(2) For queens, set diagonal elements to 1.

(3) For drones, set diagonal elements to ½.

(4) For queen *i* with dam d and sire s, element j of row i ($A_{\mathrm{ij}}$) in **A** is computed as:

$A_{ij}=\frac{1}{2}A_{dj}+A_{sj}$, for j=1, 2, ..., *i*-1.

(4.1) Elements in column *i* are obtained by symmetry.

(4.2) Add $A_{ds}$ to $A_{ii}$.

(5) For drone *i* with dam *d*, element $A_{ij}$ is computed as:

$A_{ij}=\frac{1}{2}A_{dj}$ for *j*=1, 2, ... , *i*-1.

(5.1) Elements in column *i* are obtained by symmetry.

Given the high number of drones and queens present in the relationship matrix, there was a need to vectorize calculations to drastically accelerate computations. When transcribed in a R functional algorithm (R Core team, 2017), step 4 and 5 were therefore vectorized and symmetric values obtained through transpositions. We used packages **pryr** (Hadley Wickham, 2018) and **lineprof** (sub package of Hadley Wickham available on his GitHub directory) to check for correct vectorization interpretations.
