# Supplementary material for: A simulation study of a honeybee breeding scheme accounting for polyandry, direct and maternal effects on colony performance
Source: Genet Sel Evol. 2021 Sep 8;53:71. doi: 10.1186/s12711-021-00665-8 (PMC8425095; doi:10.1186/s12711-021-00665-8)
Supplement: Supplementary file 3 — Additional file 3. R Packages used for programming. List of R packages used in the simulation program. [file 12711_2021_665_MOESM3_ESM.docx]

# Additional file 3

## **R Packages used for programming**

The language used for programming is R (R Core team, 2017), supported by the Foundation for Statistical Computing, Vienna, Austria. URL: https://www.r-project.org/

The simulation script was entirely written de novo and relies on the use of the following R packages:

**dplyr**: Hadley Wickham, Romain François, Lionel Henry and Kirill Müller (2018). dplyr: A Grammar of Data Manipulation. R package version 0.8.0.1.

**tidyr**: Hadley Wickham and Lionel Henry (2019). tidyr: Easily Tidy Data with 'spread()' and 'gather()' Functions. R package version 0.8.3.

**data.table**: Matt Dowle and Arun Srinivasan (2019). data.table: Extension of `data.frame`. R package version 1.12.2.

It can also use **BLUPf90** (Misztal *et al.* 2002) for Blup indexes predictions (option not used in this study).
